# Integrated analysis of multimodal long-read epigenetic assays

**DOI:** 10.1101/2025.11.09.687458

**Published:** 2026-01-14

**Authors:** Jeremy Marcus, Oberon Dixon-Luinenburg, Nathan Gamarra, Jacob P. Schwartz, Michal Rozenwald, Annie Maslan, Fyodor D. Urnov, Aaron F. Straight, Nicolas Altemose, Nilah M. Ioannidis, Aaron Streets

## Abstract

Long-read sequencing assays that detect base modifications are becoming increasingly important research tools for the study of epigenetic regulation, especially with the development of DiMeLo-seq and similar methods that deposit non-native base modifications to mark a range of epigenetic features such as protein-DNA interactions and chromatin accessibility. A main benefit of these methods is their inherent capacity for multimodality, enabling the encoding of multiple genomic signals onto single nucleic acid molecules. However, there are limited tools available for visualization and statistical analysis of this type of multimodal data. Here we introduce *dimelo-toolkit* , a python package built to enable flexible visualizations and easy integration into custom data processing workflows. We demonstrate the utility of *dimelo-toolkit* ’s preset visualizations of multiple base modifications in long-read single-molecule sequencing data with a novel extension of the DiMeLo-seq protocol that can capture three separate aspects of chromatin state on the same single reads: target protein binding, CpG methylation, and chromatin accessibility. We apply this multimodal method to simultaneously map chromatin accessibility, CpG methylation, and LMNB1 and CTCF binding patterns, respectively, in GM12878 cells. Our flexible design allows us to investigate previously unexplored technical biases that arise when working with this type of multimodal data. Additionally, we show that *dimelo-toolkit* enables analysis for a wide range of other long-read sequencing methods, such as mapping endogenous patterns in RNA base modifications with direct RNA sequencing. This software tool will pave the way for developing well-optimized protocols and help unlock previously inaccessible biological insights.

## Full Text Availability

The license terms selected by the author(s) for this preprint version do not permit archiving in PMC. The full text is available from the preprint server.

