# Supplementary material for: Integrated analysis of multimodal long-read epigenetic assays
Source: bioRxiv. 2025 Nov 9:2025.11.09.687458. Preprint. [Version 1] doi: 10.1101/2025.11.09.687458 (PMC12637566; doi:10.1101/2025.11.09.687458)
Supplement: Supplementary file 1 [file NIHPP2025.11.09.687458V1-supplement-1.pdf]

bioRxiv preprint doi: https://doi.org/10.1101/2025.11.09.687458; this version posted November 9, 2025. The copyright holder for this preprint (which was not certified by peer review) is the author/funder, who has granted bioRxiv a license to display the preprint in perpetuity. It is made available under aCC-BY 4.0 International license.

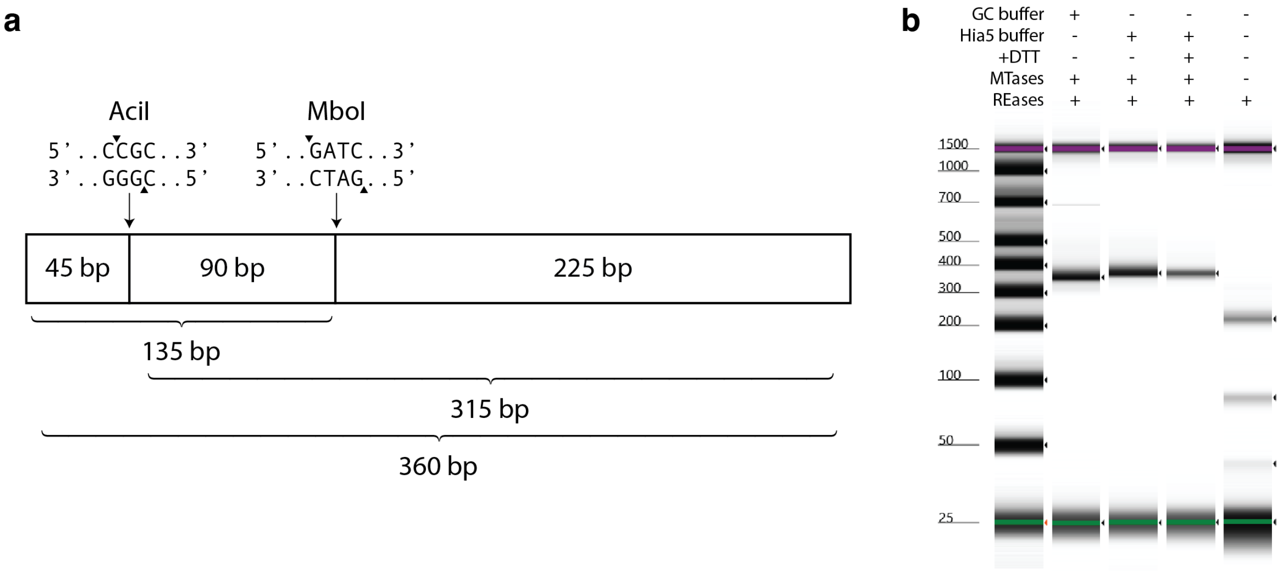

Supplementary Figure 1. Validation of methyltransferase function using restriction enzyme digestion. a, *Diagram of DNA template used for digestion assay. A 360 bp long unmethylated template was designed to contain a single restriction site each for AciI and MboI. AciI activity is blocked by cytosine methylation and MboI activity is blocked by adenine methylation. Restriction sites were placed such that unique combinations of methylation and digestion events result in fragments of unique lengths.* ***b,*** *Restriction enzyme digestion experiment testing methylation buffer conditions. DNA template from* ***(a)*** *was incubated with both M.CviPI and pA-Hia5 for 2 hours in different buffers, then subjected to restriction digest by AciI and MboI. GC buffer is NEB GC Reaction Buffer supplied with M.CviPI. Hia5 buffer is standard DiMeLo-seq methylation buffer. +DTT represents the addition of 10mM DTT. MTases and REases represent the addition of methyltransferases and restriction enzymes respectively.*

bioRxiv preprint doi: https://doi.org/10.1101/2025.11.09.687458; this version posted November 9, 2025. The copyright holder for this preprint (which was not certified by peer review) is the author/funder, who has granted bioRxiv a license to display the preprint in perpetuity. It is made available under aCC-BY 4.0 International license.

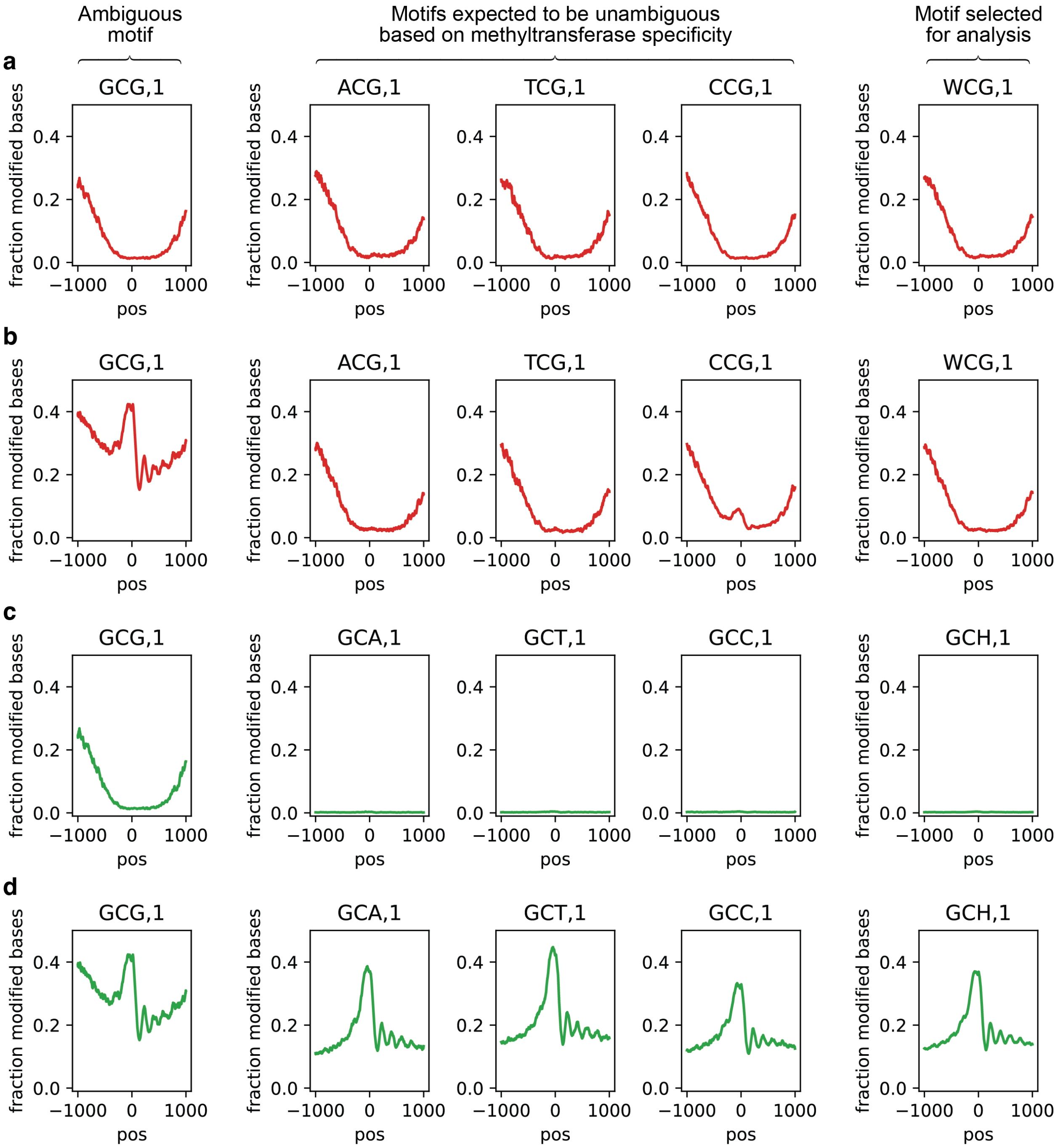

Supplementary Figure 2. Motif disambiguation for 5mC detection in the presence of exogenous GpC methylation. a-b*, CpG methylation profiles (red) for high-expression TSSs, at CG motifs with different preceding bases, in untreated* ***(a)*** *and M.CviPI-treated* ***(b)*** *conditions.* ***c-d****, GpC methylation profiles (green) for high-expression TSSs, at GC motifs with different succeeding bases, in untreated* ***(c)*** *and M.CviPI-treated* ***(d)*** *conditions. All profiles were generated using a modification probability threshold of 0.75 and a 25 bp rolling window. Subplot titles represent the motif codes used for each pileup operation. Left column represents the GCG motif which is shared between CG and GC methyltransferases. Middle columns represent*

bioRxiv preprint doi: https://doi.org/10.1101/2025.11.09.687458; this version posted November 9, 2025. The copyright holder for this preprint (which was not certified by peer review) is the author/funder, who has granted bioRxiv a license to display the preprint in perpetuity. It is made available under aCC-BY 4.0 International license.

motifs which are not expected to be shared between the methyltransferases. Right column represents the disambiguated motif selected for the analyses in this study.

bioRxiv preprint doi: https://doi.org/10.1101/2025.11.09.687458; this version posted November 9, 2025. The copyright holder for this preprint (which was not certified by peer review) is the author/funder, who has granted bioRxiv a license to display the preprint in perpetuity. It is made available under aCC-BY 4.0 International license.

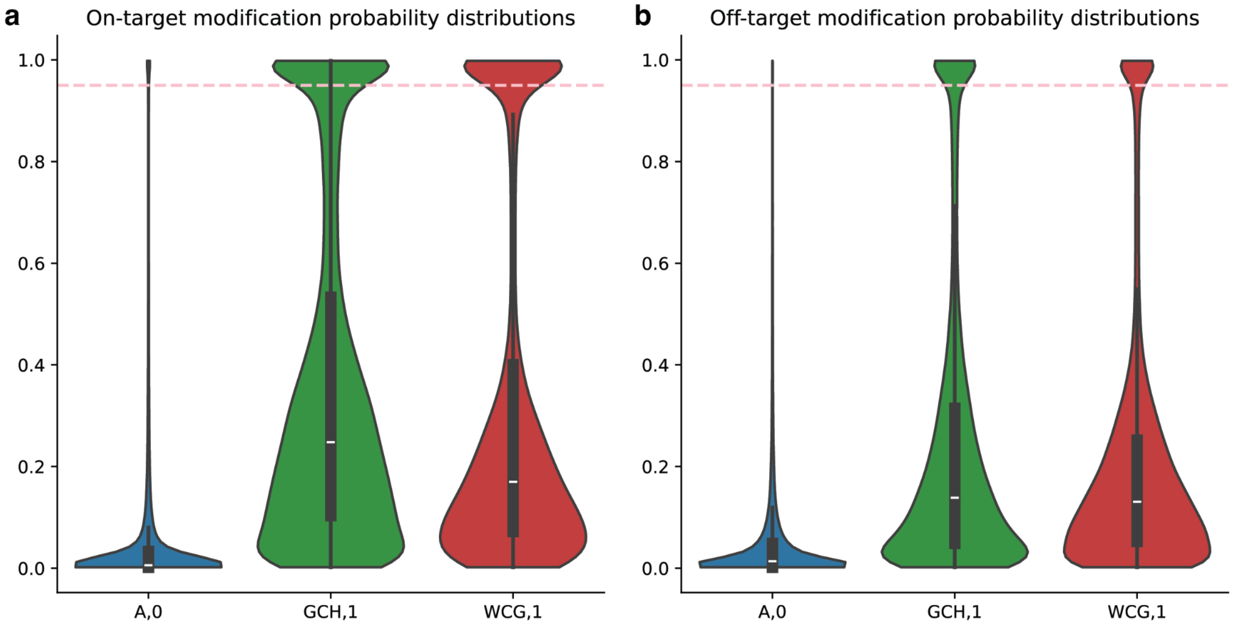

Supplementary Figure 3. Modification probability distributions in LMNB1-targeting multimodal DiMeLo-seq experiment in GM12878 cells. a-b, *Distributions of modified base probabilities reported by the dorado basecaller for 6mA (blue; targeted to LMNB1), 5mC in GC motifs (green; accessible chromatin), and 5mC in CG motifs (red; endogenous CpG methylation). The pink dashed line represents the modification probability threshold of 0.95 selected for subsequent analysis of this data. Distributions are shown for on-target regions* ***(a)*** *and off-target regions* ***(b)*** *for each methylation signal. On- and off-target regions are defined the same way as in Figure 2: for 6mA, on-target regions are cLADs and off-target regions are ciLADs. For 5mC in GC motifs, on-target regions are active TSSs and off-target regions are the flanking regions of those TSSs. For 5mC in CG motifs, on-target regions are CpG islands overlapping inactive TSSs and off-target regions are CpG islands overlapping active TSSs.*

bioRxiv preprint doi: https://doi.org/10.1101/2025.11.09.687458; this version posted November 9, 2025. The copyright holder for this preprint (which was not certified by peer review) is the author/funder, who has granted bioRxiv a license to display the preprint in perpetuity. It is made available under aCC-BY 4.0 International license.

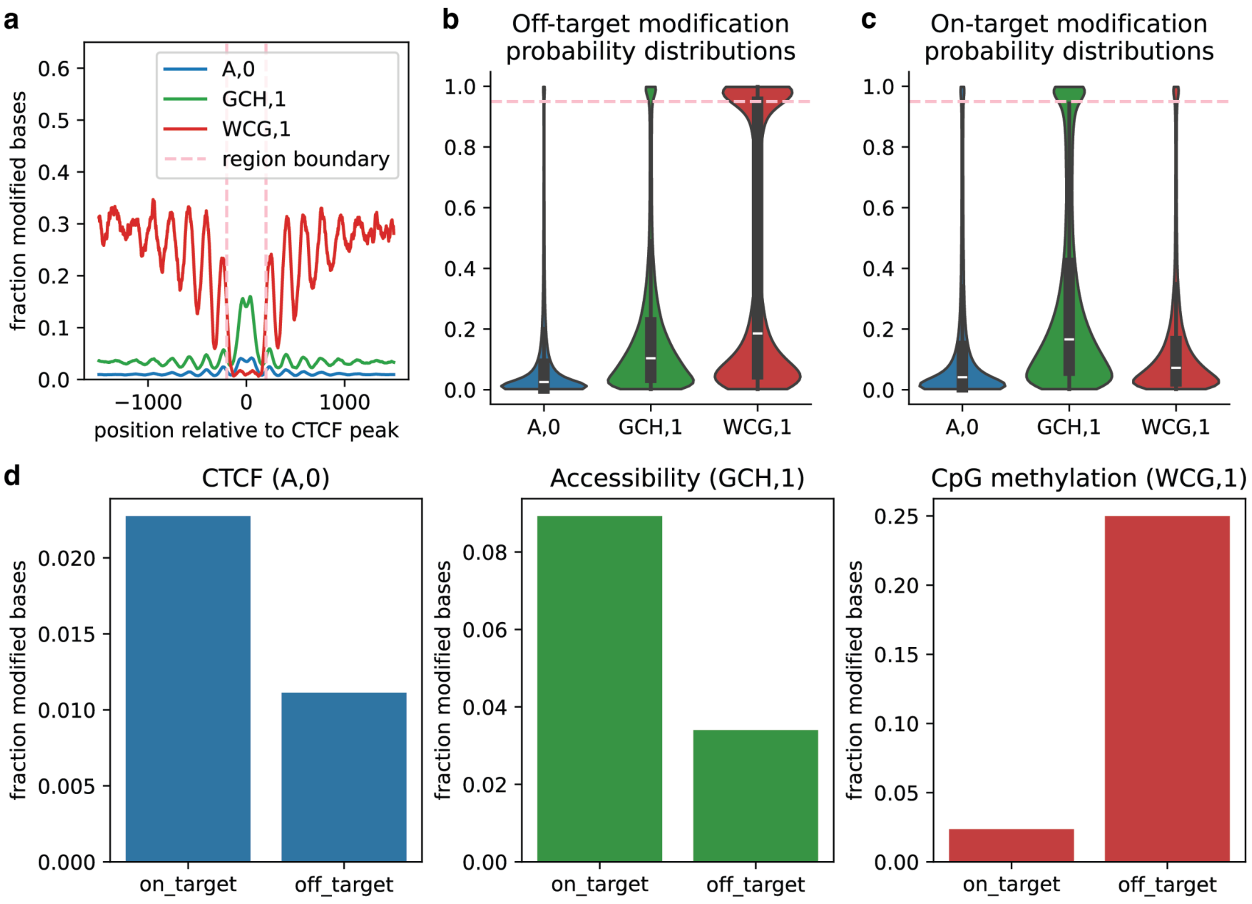

Supplementary Figure 4. Modification probability distributions in CTCF-targeting multimodal DiMeLo-seq experiment in GM12878 cells. a*, Enrichment profiles describing CTCF occupancy (blue), accessibility (green), and endogenous CpG methylation (red). Pink dashed lines represent the boundary defining on-target regions (+/- 200 bp from peak center) and off-target regions (1,300 bp flanking on-target regions on both sides).* ***b-c****, Distributions of modified base probabilities reported by the dorado basecaller for 6mA (blue; targeted to CTCF), 5mC in GC motifs (green; accessible chromatin), and 5mC in CG motifs (red; endogenous CpG methylation). The pink dashed line represents the modification probability threshold of 0.95 selected for subsequent analysis of this data. Distributions are shown in the on-target* ***(b)*** *and off-target* ***(c)*** *regions defined in* ***(a)****.* ***d****, Barplots of modified base fractions in regions defined in* ***(a)*** *for modifications reported with probability >0.95.*
